# A medical insurance fund operation performance evaluation system under the DRG payment mode reform

**DOI:** 10.3389/fpubh.2025.1549575

**Published:** 2025-02-10

**Authors:** Zijian Wang, Weifu Chang, Aijing Luo

**Affiliations:** ^1^The Second Xiangya Hospital of Central South University, Changsha, China; ^2^School of Public Administration, Central South University, Changsha, China; ^3^Key Laboratory of Medical Information Research (Central South University), College of Hunan Province, Changsha, China; ^4^The Third Xiangya Hospital of Central South University, Changsha, China; ^5^Clinical Research Center For Cardiovascular Intelligent Healthcare in Hunan Province, Changsha, China

**Keywords:** medical insurance funds, AHP-EWM-FCE methodology, performance evaluation, DRG payment reform, Tobit regression model

## Abstract

**Background:**

An evaluation system for comprehensively measuring medical insurance fund operational performance under China’s Diagnosis Related Groups (DRG) payment reform holds critical theoretical and practical significance, especially for enhancing the efficiency of medical insurance fund utilization and the quality of healthcare services. However, few studies undertake performance evaluations of medical insurance funds under DRG payments, especially those incorporating the Analytic Hierarchy Process (AHP)-Entropy Weight Method (EWM)- Fuzzy Comprehensive Evaluation (FCE) method model.

**Methods:**

This study utilizes operational data from medical insurance funds across eight cities in S Province, China, from 2020 to 2022. It develops an innovative performance evaluation system for medical insurance funds utilizing the AHP-EWM-FCE evaluation method. Finally, it explores the key influencing factors by applying the Tobit regression model.

**Results:**

As the reform of DRG payment methods has advanced, the operational performance scores related to the management, fundraising, utilization, and satisfaction of DRG medical insurance funds have consistently improved. Notably, the comprehensive indexes of QD and JN cities exhibit significant comparative advantages, resulting in higher performance evaluation scores for their medical insurance funds. Additionally, the performance scores assessed by the proposed evaluation system align closely with actual operational outcomes. Regression analysis further indicates that medical service capability is the key determinant influencing the operational performance of medical insurance funds.

**Conclusion:**

This study develops a novel evaluation system for measuring medical insurance funds’ operational performance. The insights can help proactively foster the high-quality development of these funds, and modernization of the medical insurance governance system and governance capabilities; stimulate the fund’s productivity; and enhance the health and wellbeing of people.

## Background

China has successfully established a universal basic medical insurance system. This system has achieved an impressive coverage rate of over 95% overall, which even approaches 100% in some cities (1). The urban employee medical insurance amount was raised to 5,737 yuan in 2022, while the urban–rural resident medical insurance increased to 1,029 yuan. The corresponding policy reimbursement rates stand at 84.2 and 68.3%, respectively (2). However, the current medical insurance funding system faces issues such as a large interest group in the field of medical services, information asymmetry, high technical barriers, and frequent policy risks. Consequently, a medical insurance service system that aligns with China’s unique characteristics is needed (3). Indeed, in terms of medical insurance payment model reform, China’s National Medical Security Administration has pioneered the implementation of the globally recognized scientific payment mode: DRG payment. Since 2019, this DRG payment model has been piloted in 30 cities across the country (4).

Capitalizing on the “law of large numbers” methodology, the DRG payment model offers a vital mechanism and management tool for macro-controls in modern medical insurance payment mode reforms. Here, an evaluation system for comprehensively measuring medical insurance fund operation performance, considering DRG payment, can be extremely useful in driving healthcare institutions to shift from “extensive” management to more “refined” approaches. For instance, such a performance evaluation system can promote the rational allocation of medical resources, reduce resource waste, and improve the quality and efficiency of medical insurance funds (5). In November 2021, the National Medical Security Administration released the Three-Year Action Plan for DRG/DIP Payment Mode Reform, which clearly stated that by 2025, DRG/Big Data Diagnosis-Intervention Packet (DIP) should be comprehensively implemented in four areas: overall planning, health care institutions, disease grouping, and medical insurance fund (6). This initiative aims to construct a robust health security network for the public, thereby serving as a powerful “stabilizer” for the steady progress of socialism with Chinese characteristics (7).

Still, a mature evaluation system for examining medical insurance fund operation performance is lacking both domestically and internationally. Currently, such an evaluation system considering the DRG payment mode reform has several shortcomings, including singularity, extensiveness, limitations in the dimensions and indicators considered, and the lack of comprehensive consideration for the medical insurance fund operations (8). Furthermore, due to the insufficient accumulation of historical data from the DRG payment model pilot cities in the early stages of implementation, it was unable to accurately analyze from the perspectives of institutional embedding, regulatory behavior, medical behavior, and social interaction (8). Consequently, constructing a rigorous and robust evaluation system of medical insurance fund operation performance under the DRG payment mode reform is a critical task.

The literature shows that the research focuses of foreign medical insurance funds are different. For example, Avedis Donabedian, the father of American medical management, divided medical insurance evaluation indicators into structure, process, and result evaluations, focusing on the efficiency and fairness of medical insurance fund allocation (9). British and Organisation for Economic Cooperation and Development (OECD) medical insurance emphasizes the fairness and accessibility of medical insurance but neglects efficiency evaluation (10–12). One study has used the content validity ratio to screen the identified factors to identify and rank critical factors affecting hospital performance using the best-worst method in Iran (13). In China, research on the performance evaluation system of medical insurance funds is still in the exploratory stage. An analysis of the medical insurance fund operation in Jiaxing City in 2019 used an evaluation system consisting of four dimensions and 10 indicators (14). Yu and Zhi empirically examined urban employee and rural resident basic insurance schemes in Yantai City, providing an initial overview of the status of the city’s medical insurance fund operations (15). Meanwhile, research has also evaluated the performance system of public hospitals based on single balanced scorecard theory and the AHP, thereby establishing a model for the performance system of public hospitals (16, 17). However, the above studies have certain limitations in terms of the selection of horizontal indicators, vertical time range, methodology, and empirical research. Thus, they have failed to perform a comprehensive and dynamic evaluation of the actual operation of the medical insurance fund. Therefore, a rigorous performance evaluation system that encompasses comprehensive indicators and can dynamically evaluate the medical insurance fund operation is lacking.

Naturally, one may ask: How should we construct a comprehensive and dynamic performance evaluation system for medical insurance fund operation under the DRG payment method reform? One particular area can be using more rigorous and scientific research methods, which can enhance the focus ability and objectively evaluate the dynamic running differences of medical insurance funds. Among them, AHP can carry out step-by-step analysis and comprehensive evaluation of complex decision-making problems within a structured framework, thereby making more scientific and reasonable choices (18). AHP yields subjective weights, while the EWM yields objective weights. Then, the combination of EWM and AHP can be used to obtain and assign effective and true indicator weights (19). In addition, FCE can better handle the interaction between various complex factors, especially the performance evaluation of medical insurance funds, which has uncertainty, complexity, and dynamics (20–22). However, to the best of our knowledge, no studies have examined the comprehensive use of the AHP-EWM-FCE method for the performance evaluation of the medical insurance fund operation under the backdrop of DRG payments. Addressing this gap, this research uses operational data on medical insurance funds in eight cities in Province S of China over 3 years to construct a comprehensive and dynamic performance evaluation system for the medical insurance fund operation under the DRG payment method reform.

In summary, this study applies the AHP-EWM-FCE method to evaluate the operational performance of medical insurance funds within the context of the DRG payment system. Next, utilizing 3 years of operational data from medical insurance funds across eight cities in S Province, China, this study employs Tobit regression analysis to identify the key factors influencing the performance evaluation. The aim is to develop a comprehensive and dynamic performance evaluation system for medical insurance funds within the framework of the DRG payment reform.

## Methods

### Data sources and research methods

The literature and data for this study are sourced from both domestic and international databases, including the Web of Science, PubMed, China National Knowledge Infrastructure (CNKI), Wanfang Data Knowledge Service Platform, and VIP Chinese Journal Service Database. The statistical data for the indicator system mainly come from the National Tertiary Public Hospital Performance Appraisal Operation Manual, National Healthcare Security Diagnostic Related Groups (CHS-DRG) Grouping Plan, National Medical insurance Diagnostic Related Groups (CHS-DRG) Grouping and Payment Technical Specifications, Statistical Yearbook of S Province, Health Statistics Yearbook of S Province, Statistical Yearbook of Population and Employment Statistics of S Province, Statistical Yearbook of Civil Affairs of S Province, Social Statistics Yearbook of S Province, performance assessment data from some hospitals in eight cities of S province, and information from official hospital websites. To ensure the richness and authenticity of the data, field visits and semi-structured interviews were carried out to gather first-hand interview materials.

### Preliminary determination of medical insurance fund indicators using the Delphi method

#### Expert selection

This study employs the Delphi expert consultation method. First, expert inquiry forms are disseminated via Questionnaire Star to recruit experts. Then, consultations or interviews with selected theorists and field experts affiliated with the domain of medical insurance fund operation management (such as medical insurance bureaus, health commissions, hospital medical insurance offices, and clinical medical staff) are conducted. Aggregating diverse viewpoints and professional knowledge from this expert sample ensures the professionalism and practicality of constructing evaluation models, and elicits more scientifically valid evaluation indicators and suggestions.

### Construction of the performance evaluation system

#### Establishment of subjective weights for performance evaluation indicators by AHP

This study utilizes AHP to delineate the subjective weights of the constructed performance evaluation indicators via four main steps: building a hierarchical model, constructing a judgment matrix, calculating indicator weights, and testing consistency. Meanwhile, the concentration of expert opinions is measured by the mean and full score rate, while the coordination of expert opinions is measured via the dispersion coefficient (23, 24). Finally, the resulting index system is subjected to reliability and validity tests.

#### Establishment of objective weights for performance evaluation indicators by EWM

Operating data on 28 medical insurance fund operation performance indicators across eight sampled cities are used to assess matrix entropy values, and the randomness and disorder of the indicators. Next, the entropy value is employed to determine the degree of dispersion among the indicators. The degree of variation in the indicators is ascertained based on their weights, thereby eliminating subjective interference. EWM is subsequently used to determine the objective weights of each evaluation factor. To avoid the impact of singularity on the results of FCE, the subjective and objective weights of AHP-EWM are reconstructed using the geometric mean method to obtain new combined weights (25).

### FCE evaluation

#### Determining evaluation factors and membership evaluation criteria

The comprehensive evaluation results are divided into five levels, each corresponding to different judgment values. Simultaneously, to obtain more authoritative results, 10 experts are invited for scoring. The membership degree of evaluation factors to comment level is determined through expert scoring and percentage statistics. The fuzzy evaluation set is then calculated by combining the membership and matrix.

#### Fuzzy evaluation of medical insurance performance results

**Figure 1:**
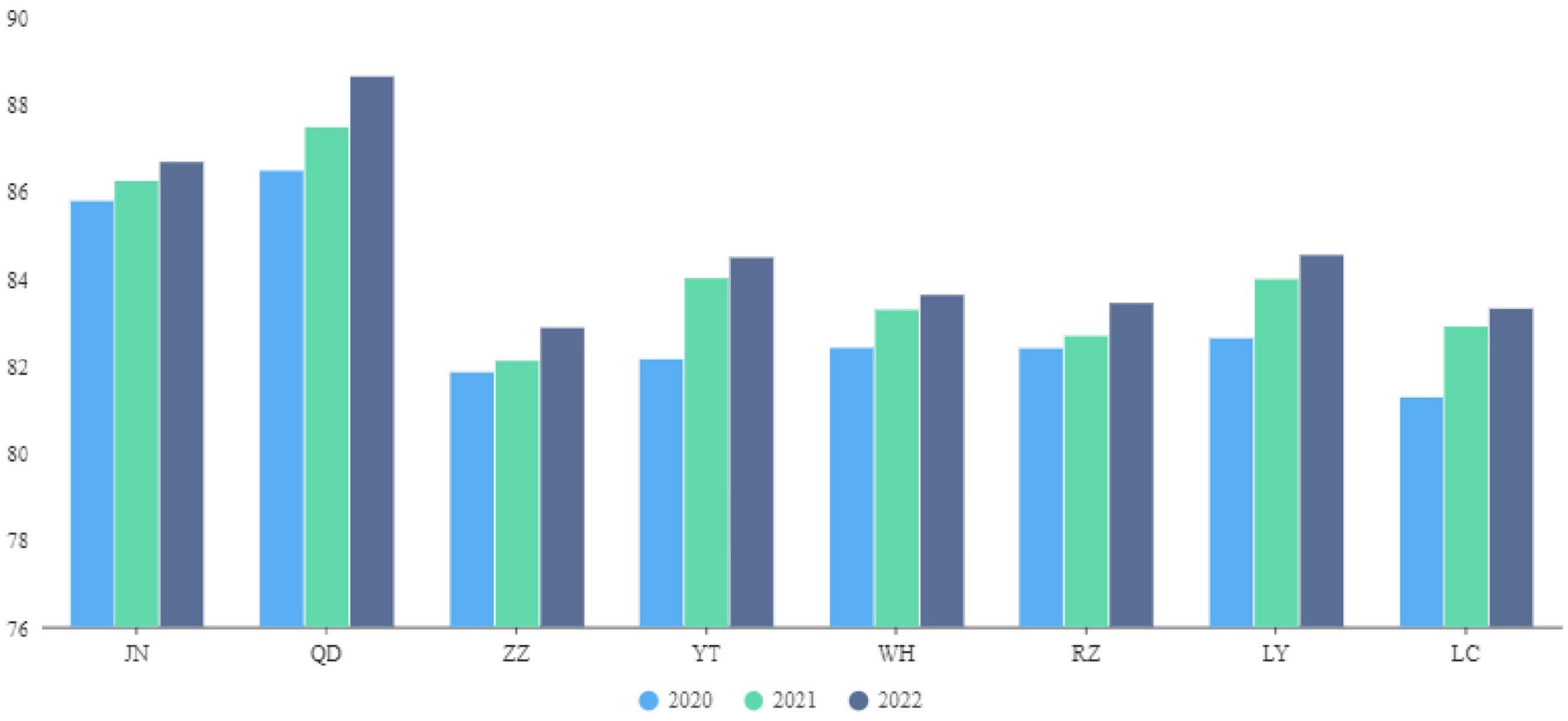
Horizontal and vertical comparisons of the performance of medical insurance fund.

As noted above, the fuzzy evaluation result is the product of membership and matrix, *C=B·V^T^*, which produces the fuzzy comprehensive evaluation of each criterion layer’s corresponding indicator (26). This outcome allows for horizontal and vertical comparisons of the performance of the medical insurance fund in eight cities in S Province from 2020 to 2022 (Figure 1). Horizontal comparison refers to assessing the performance of the medical insurance fund and vertical comparison involves examining the performance of the medical insurance fund during 2020–2022. Next, the following Tobit regression model is employed to analyze the key factors influencing the performance evaluation.


$${Y_{i}}^{*}=\beta_{0}+{\overset{k}{\sum }}_{j=1}\beta_{j}X_{kj}+\varepsilon_{i}$$

$$Y_{i}={y_{i}}^{*},\mathrm{if}\;{y_{i}}^{*}\in \left(0,+\infty \right)$$

$$Y_{i}=0,\mathrm{if}\;{yi}^{*}\in \left(-\infty ,0\right)$$

Here, the latent variable is expressed as Yi*.

The evaluation scores derived from the AHP-EWM-FCE method for the eight municipalities in S Province are used as the dependent variables, while eight secondary operational indicators serve as the independent variables. Stata 17.0 software has been used for the regression analysis.

## Results

This study preliminarily constructs a comprehensive pool of 44 potential indicators through literature review and expert consultation. Then, through an in-depth analysis of correlations and importance of these indicators, those with low correlation and high redundancy are eliminated. Simultaneously, new indicators intuitively reflecting medical insurance fund performance evaluation are introduced. Ultimately, 33 core indicators have been carefully selected and integrated after expert inquiry. And the 33 core indicators were further verified through reliability and validity tests with their validity and operability. We subject this evaluation indicator system to the empirical case of the eight cities in S Province in China. Finally, the Tobit regression model is employed to analyze the key factors influencing the performance evaluation.

### Determining performance evaluation indicators using the Delphi method

#### Basic information about experts

This study has engaged 28 experts from domestic universities, medical institutions, and medical insurance management departments. The experts are between 30 to 65 years old, with 90.00% holding a bachelor’s degree or above. In the two rounds of expert consultation, 28 and 22 questionnaires have been distributed, respectively, yielding effective recovery rates of 92.86 and 95.45%, respectively. The questionnaire recovery rate for both rounds is 94.00%, indicating that the experts have exhibited substantial enthusiasm in participating in the study.

#### Expert authority and opinion coordination degree

Table 1 shows that the authority coefficient of the experts is 0.865, indicating that this indicator aligns with the established standards. This supports the professionalism and authority of the evaluation process. The degree of expert coordination is depicted by Kendall’s W coordination coefficient. In the two rounds, the *p*-value (<0.001) for the third-level indicators showed reliable results.

**Table 1:** Degree of expert consultation coordination.

	First round			Second round		
	*W*-value	χ^2^-value	*p*-value	W-value	χ^2^-value	*P*-value
First-level indicators and weights	0.136	8.561	0.036	0.17	13.286	0.004
Second-level indicators and weights	0.117	17.154	0.016	0.085	15.549	0.03
Third-level indicators and weights	0.052	29.648	0.33	0.089	73.696	<0.001

After two rounds of expert consultation, the first-level indicators include medical insurance fund management, medical insurance fund raising, DRG operation and use of medical insurance fund, and satisfaction with medical institutions. Second-level indicators include medical insurance fund policy, platform construction, medical insurance fund raising, expenditure and balance, medical service capability, expense control, reimbursement, and satisfaction with medical institutions. Finally, 28 indicators exist in the third level, including the number of participants in basic medical insurance, financial subsidy for resident medical insurance, growth rate of medical insurance fund income, proportion of recovery of medical insurance funds, growth rate of total fund expenditure over the previous year, current balance rate of medical insurance fund, number of months supported by the medical insurance fund balance and so on. Further the DRG group number refers to a diseases classification system and the number of diseases based on clinical symptoms.

### Construction of performance evaluation system

#### Determination of AHP weight

The composite weights are then calculated using AHP. The highest weight is for the medical insurance fund information management at 0.1265. Satisfaction of medical staff and patients in medical institutions with the medical insurance fund system have weights of 0.1216 and 0.1204, respectively. The weights for financial subsidy for resident medical insurance, number of participants in basic medical insurance, growth rate of medical insurance fund income, and medical insurance fund participation rate are 0.052, 0.052, 0.0514, and 0.0514, respectively. Next, the proportion of recovery of medical insurance funds, medical insurance supervision and risk control measures, and where there is any publicity and education work on the medical insurance fund system have weights of 0.0499, 0.0418, and 0.0414, respectively (Table 2).

**Table 2:** Weight of indicators at each level.

First-level indicator	First-level weight	Second-level indicator	Second-level weight	Third-level indicator	Third-level weight	Combination weight
A1 Medical insurance fund management	0.2518	B1 Medical insurance fund policy	0.4975	C1 Whether to establish a medical insurance fund system	0.3366	0.0422
				C2 Whether there is medical insurance fund system publicity	0.3300	0.0414
				C3 Medical insurance supervision and risk control measures	0.3333	0.0418
		B2 Medical insurance fund service platform construction	0.5025	C4 Medical insurance fund information management	1.0000	0.1265
A2 Medical insurance fund raising	0.2567	B3 Medical insurance fund raising	1.0000	C5 Number of participants in basic medical insurance	0.2024	0.0520
				C6 Medical insurance fund participation rate	0.2004	0.0514
				C7 Financial subsidy for resident medical insurance	0.2024	0.0520
				C8 Growth rate of medical insurance fund income	0.2004	0.0514
				C9 Proportion of recovery of medical insurance funds	0.1944	0.0499
A3 DRG operation and use of medical insurance fund	0.2494	B4 Medical insurance fund expenditure and balance	0.2530	C10 Growth rate of total fund expenditure over the previous year	0.3232	0.0204
				C11 Current balance rate of medical insurance fund	0.3333	0.0210
				C12 Number of months of medical insurance fund balance support	0.3434	0.0217
		B5 Medical service capability	0.2555	C13 DRG group number	0.1231	0.0078
				C14 Case Mix Index (CMI)	0.1256	0.0080
				C15 Number of diseases	0.1256	0.0080
				C16 Total weight value (number of discharged patients and number of operations)	0.1281	0.0082
				C17 Average length of stay	0.1256	0.0080
				C18 Implementation of hierarchical diagnosis and treatment	0.1244	0.0079
				C19 Low risk average mortality rate	0.1231	0.0078
				C20 Number of hospitalizations	0.1244	0.0079
		B6 Medical expense control	0.2457	C21 Average hospitalization cost increase	0.3300	0.0202
				C22 Average outpatient cost increase	0.3267	0.0200
				C23 Proportion of medical service revenue	0.3432	0.0210
		B7 Medical insurance fund reimbursement	0.2457	C24 Off-site reimbursement rate	0.3333	0.0204
				C25 Inter-provincial direct settlement rate for out-of-town hospitalization expenses	0.3333	0.0204
				C26 Timely reimbursement rate of medical insurance fund	0.3333	0.0204
A4 Satisfaction	0.2421	B8 Satisfaction with medical institutions	1.0000	C27 Satisfaction of medical staff in medical institutions with medical insurance fund system	0.5025	0.1216
				C28 Satisfaction of patients in medical institutions with medical insurance fund system	0.4975	0.1204

#### Determination of EWM weight

Next, EWM is employed to construct the judgment matrix and calculate the information entropy of the comprehensive evaluation indicators to determine objective weights. The highest weight for the proportion of recovered medical insurance funds is 0.1752. The weights for total weight value (number of discharged patients and number of operations), number of diseases, and number of hospitalizations are 0.1264, 0.1200, and 0.1002, respectively. The weights for number of participants in basic medical insurance, current balance rate of medical insurance fund, number of months of medical insurance fund balance support, inter-provincial direct settlement rate for out-of-town hospitalization expenses, and off-site reimbursement rate are 0.0648, 0.0603, 0.0566, 0.0416, and 0.0391, respectively (Table 3).

**Table 3:** Combined weight of third-level indicators.

Third-level indicators	AHP weight	EWM weight	Combined weight
C1 Whether to establish a medical insurance fund system	0.0422	0.0007	0.0082
C2 Whether there is medical insurance fund system publicity	0.0414	0.0008	0.0089
C3 Medical insurance supervision and risk control measures	0.0418	0.0008	0.0088
C4 Medical insurance fund information management	0.1265	0.0025	0.0276
C5 Number of participants in basic medical insurance	0.0520	0.0648	0.0891
C6 Medical insurance fund participation rate	0.0514	0.0016	0.0140
C7 Financial subsidy for resident medical insurance	0.0520	0.0351	0.0656
C8 Growth rate of medical insurance fund income	0.0514	0.0388	0.0687
C9 Proportion of recovery of medical insurance funds	0.0499	0.1752	0.1436
C10 Growth rate of total fund expenditure over the previous year	0.0204	0.0220	0.0325
C11 Current balance rate of medical insurance fund	0.0210	0.0603	0.0547
C12 Number of months of medical insurance fund balance support	0.0217	0.0566	0.0538
C13 DRG group number	0.0078	0.0097	0.0134
C14 Case Mix Index (CMI)	0.0080	0.0193	0.0191
C15 Number of diseases	0.0080	0.1200	0.0476
C16 Total weight value (number of discharged patients and number of operations)	0.0082	0.1264	0.0493
C17 Average length of stay	0.0080	0.0106	0.0141
C18 Implementation of hierarchical diagnosis and treatment	0.0079	0.0000	0.0009
C19 Low risk average mortality rate	0.0078	0.0091	0.0130
C20 Number of hospitalizations	0.0079	0.1002	0.0433
C21 Average hospitalization cost increase	0.0202	0.0376	0.0424
C22 Average outpatient cost increase	0.0200	0.0130	0.0248
C23 Proportion of medical service revenue	0.0210	0.0098	0.0221
C24 Off-site reimbursement rate	0.0204	0.0391	0.0434
C25 Inter-provincial direct settlement rate for out-of-town hospitalization expenses	0.0204	0.0416	0.0448
C26 Timely reimbursement rate of medical insurance fund	0.0204	0.0019	0.0096
C27 Satisfaction of medical staff in medical institutions with medical insurance fund system	0.1216	0.0013	0.0192
C28 Satisfaction of patients in medical institutions with medical insurance fund system	0.1204	0.0011	0.0174

#### Determination of AHP-EWM combined weights

The geometric average method is used to reconstruct the AHP subjective and EWM objective weights, highlighting the importance of weights and disparity after reconstruction to obtain the combined weights. The weight of proportion of recovery of medical insurance funds is the highest at 0.1436. Next, the weights for number of participants in basic medical insurance, growth rate of medical insurance fund income, financial subsidy for resident medical insurance, current balance rate of medical insurance fund, and number of months of medical insurance fund balance support are 0.0891, 0.0687, 0.0656, 0.0547, and 0.0538, respectively (Table 3).

#### Evaluation index system construction and empirical research

Next, this article selects some designated medical institutions in the eight DRG payment pilot cities of S Province as case studies for the empirical research. According to the AHP-EWM-FCE evaluation results, the performance score of the medical insurance fund operation exhibits an upward trend across the sampled cities. The scores for JN and QD are relatively high between 2020 and 2022 at 85.76, 86.21, and 86.66, respectively, for JN, and 86.46, 87.46, and 88.62, respectively, for QD (Figure 1).

The Tobit regression analysis reveals that the two indices are significantly related to the medical insurance fund’s operational performance. Specifically, medical service capacity, medical insurance fund raising, and expenditure and balance of the medical insurance fund exhibit a significant positive coefficient with the operational performance of the medical insurance fund (Table 4).

**Table 4:** Tobit regression analysis results for influencing factors.

Second-level indicator	Regression coefficient	Standard deviation	*T-*value	*P*-value	Sig.
Medical insurance fund policy	0.033	1.34e-08	2440040.93	0	***
Construction of medical insurance fund service platform	0.018	1.88e-08	952141.98	0	***
Medical insurance fund raising	0.287	1.09e-08	26232752.12	0	***
Expenditure and balance of medical insurance fund	0.217	1.50e-08	14518224.56	0	***
Medical service capacity	0.401	1.88e-08	21318847.42	0	***
Medical cost control	0.102	7.31e-09	13952284.09	0	***
Medical insurance fund reimbursement	0.126	1.26e-08	9956651.29	0	***
Satisfaction of medical institutions	0.032	1.60e-08	2002835.78	0	***

***p < 0.01, **p < 0.05, and *p < 0.1.

## Discussion

In May 2019, QD of S Province was selected as a pilot city among the initial batch of 30 cities by the National Healthcare Security Administration to implement DRG payment reforms. In September of the same year, the Provincial Healthcare Security Administration of S Province was among the first to initiate DRG payment method reforms in 10 pilot cities in stages. However, extant research on the performance evaluation of medical insurance fund operation in the backdrop of the DRG payment method reforms has certain limitations in terms of indicator selection, time period, and methodology. Addressing this gap, this study draws on the AHP-EWM-FCE method to construct a rigorous evaluation system for examining the medical insurance fund operation performance under the DRG payment method reform over the 2020–2022 in S Province. It also performs an empirical case study using data from mainly large-scale tertiary general hospitals, thereby appropriately reflecting the diverse situations from hospital operation, referral, to off-site reimbursement, among others. Thus, the relevant conclusions offer valuable practical guidance.

### Evaluation system indicators

Extant research shows that establishing comprehensive performance evaluation indicators for the medical insurance fund operation can not only better reflect the operations, but also promote the efficient use of medical insurance funds and improvement of medical service levels (27). Accordingly, this study combines extant research and the actual operation of DRGs to establish 44 potential indicators of medical insurance fund operation. This number is reduced to 28 after two rounds of expert consultation and correction. Among them, the number of participants in basic medical insurance, financial subsidy for resident medical insurance, growth rate of medical insurance fund income, proportion of recovered medical insurance funds, growth rate of total fund expenditure over the previous year, current balance rate of the medical insurance fund, and number of months supported by the medical insurance fund balance are closely related to the efficiency and effectiveness of the use of medical insurance funds. The number of DRG groups, CMI, the number of diseases, and the total weight value (number of discharged patients and volume of surgeries) are closely related to the reform of the DRG payment method. Therefore, the selected evaluation indicators can comprehensively reflect the medical insurance fund operation under the DRG payment method.

### Indicator weights

This study is the first to use the AHP-EWM-FCE method to establish the weights of performance evaluation indicators for the medical insurance fund operation in the background of the DRG payment method reform. Among the indicators, the AHP-EWM combined weight of DRG operation and use of medical insurance fund is 0.529, the highest among all weights. This indicates that DRG operation and the use of medical insurance funds are closely related to the performance evaluation of medical insurance fund operation. According, efforts can focus on further improving the efficiency of medical insurance fund use, and standardize hospital diagnosis and treatment behaviors based on the indicators of DRG operation and use of medical insurance funds (28).

Next, the combined weight of the medical insurance fund raising indicator is 0.381, representing a relatively high weight. Research shows that an appropriate level and structure of financing for the medical insurance fund is key to promoting its accessibility, fairness, and sustainability (29). At present, China’s medical insurance fund largely depends on government subsidies, which is increasing annually (30). In recent years, Chinese scholars have explored the improvement of the medical insurance fund financing mechanism and sustainability of funds. They suggest modifications to the frequency, timing, and extent of medical insurance fund raising, alongside the establishment of actuarial models to achieve “basic, bottom-line, and sustainable” medical insurance funds (31). As such, the government and relevant medical insurance departments should establish institutionalized and standardized dynamic adjustment mechanisms for medical insurance fund raising.

Next, experts demonstrate a high degree of recognition of “medical insurance informatization.” This can assist in connecting medical information within designated medical insurance institutions and the real-time acquisition of patients’ medical insurance information. On the one hand, such efforts can facilitate the real-time monitoring of big data pertaining to medical insurance funds, and help promptly detect and correct phenomena such as the abuse of medical insurance funds, medical misconduct, and unreasonable medical expenses. On the other hand, it can aid in the promotion of hierarchical diagnosis and treatment (3). Further, the DRG payment mode reform requires accurate accounting of disease costs, which also necessitates the support of hospital information system. For instance, based on big data, Liu conducted an in-depth analysis of disease costs using the principles of Boston Matrix Analysis to identify the root cause of cost controls. Therefore, medical insurance informatization is conducive to the efficient use of medical insurance funds and refined implementation of the DRG payment reform (32).

### Empirical analysis

The performance score of the medical insurance fund operation in the eight sampled cities shows a clear growth trend over the 2020–2022 period. Among them, QD has embedded the DRG payment system into the reconstruction of its incentive structure.

### Medical insurance fund raising

Among the third-level indicators, the participants in basic medical insurance of QD and LY remain at a high level, and the medical insurance fund participation rates of JN, QD, ZZ, YT, LY, and LC all exceed 96%. Notably, the overall insurance coverage level in the eight cities exceeds the national average. This may be related to the success of the medical insurance policy promotion and universal medical insurance expansion. However, China currently adopts a voluntary participation approach for basic medical insurance. Some new employees are missing from the scheme, while coverage for workers in small and micro enterprises is not yet complete. Therefore, there is room for improvement in medical insurance coverage (33). Thus, future efforts focus on increasing publicity by the government and medical insurance companies to reduce missing coverage, increase the coverage rate of micro and small enterprises, and achieve universal medical insurance.

### Analysis of DRG operation and medical insurance fund use

#### Expenditure and balance of medical insurance funds

Under the DRG payment reform, the long-term benefits provided by the medical insurance fund to insured patients determine the integration and coordination between the medical insurance system and high-quality hospital development (34–36). In this study, JN and QD score the highest in the use of medical insurance funds. However, there is a relatively prominent imbalance in the revenue and expenditure of medical insurance funds in the eight cities. At present, China’s medical insurance fund management adopts the principle of “vertical accumulation and actuarial balance.” The imbalance between fund expenditures and balances, whether too high or too low, is not conducive to the functioning of medical insurance funds. According to Jia, the number of months for the balance payment of the medical insurance fund is an important factor its sustainability, and 6–9 months being an appropriate number (37). Specifically, if the medical insurance fund balance is paid for more (less) than nine (six) months, the fund accumulation is excessive (too low).

Meanwhile, the balances of eight cities in S Province are too small, indicating that the ability of medical insurance funds to resist risks is weak. Still, there was no cumulative deficit. Yet, the number of balance payment months in most cities is 0–1 month, and only a few cities have a balance of more than 1 month. This may be related to the fact that in the face of public emergencies in recent years, the National Medical Insurance Administration has promptly adjusted payment policies, expanded the scope of medical insurance payment, and dynamically adjusted payment and budget (38). Hence, the regulation of medical insurance funds is crucial. It is recommended to conduct horizontal risk control, avoid rough expenditures, and reduce the accumulation or deficit of medical insurance funds, thus promoting the smooth and efficient development of fund operations (39). Currently, most countries adopt diversified financing methods to balance the fund expenditures and balances (40). Irregularities and misuse of funds can be prevented by establishing effective monitoring mechanisms and auditing processes,

#### Medical service capabilities

The service capabilities of medical institutions can somewhat reflect the management level of medical insurance funds, governments, and medical institutions in the region. The survey sample comprises tertiary-A general hospitals, wherein the number of DRG groups in JN, QD, ZZ, and YT cities is relatively complete. Meanwhile, the number of DRG groups in RZ, LY, and LC is slowly increasing, indicating that the latter’s DRG groups are becoming complete (41). The CMI values of QD and JN are higher than those of other cities, indicating that they have a relatively large number of highly weighted medical records. Compared with 2020, 70.5% of hospital departments significantly increased their CMI values in 2022. Thus, the tertiary-A general hospitals accurately position themselves in diagnosing and treating difficult cases. Moreover, the average length of stay in RZ and LY has dropped to 6.14 and 6.77, reflecting good control of hospital stays by medical institutions. However, shorter hospital stays do not necessarily mean better outcomes. For instance, blindly reducing hospital stays, and refusing difficult and complex patients will lead to a decrease in the CMI value (42).

#### Medical insurance fund reimbursement

Population mobility triggers off-site reimbursements from medical insurance funds. Owing to reasons such as education, employment, or relocation, young and transient populations might travel elsewhere. These groups are more likely to engage in activities like seeking medical treatment off-site (43). Indeed, the reimbursement indicators of medical insurance funds in the eight sampled cities in S Province have shown a significant increase. QD and RZ operate well in off-site reimbursements, with some areas achieving same-day reimbursement. Meanwhile, S Province continues to promote the national network of basic medical insurance and direct settlement of hospitalization expenses across provinces and places, which greatly facilitates the efficient use of medical insurance funds. Efforts should be made to increase the rate of same-day reimbursement off-site, which can not only meet the service needs of off-site medical treatment but also increase population mobility (44).

## Conclusion

Based on operational data on medical insurance funds from eight cities in S province in China, this study comprehensively and dynamically evaluates the performance of the medical insurance fund under the DRG payment reform by constructing a rigorous performance evaluation system. This system can be used to improve the efficiency of using medical insurance funds and elevating the standard of medical services. This study is also the first to construct the evaluation system based on the AHP-EWM-FCE method. It identifies key indicators for the medical insurance fund operation, such as the DRG operation, use and management of medical insurance funds, fundraising, and satisfaction levels. Here, DRG operation and the use of medical insurance funds have the highest weights in the evaluation system. Furthermore, the performance of the proposed evaluation system is consistent with the actual operation and performance scores of the medical insurance fund across the eight cities. Overall, the proposed addresses the deficiencies in methodology, indicator system construction, and practical application with greater rigor, applicability, and promotability.

## Data Availability

The original contributions presented in the study are included in the article/supplementary material, further inquiries can be directed to the corresponding author.
